# Prognostic Value of Cavernous Sinus Invasion in Patients with Nasopharyngeal Carcinoma Treated with Intensity-Modulated Radiotherapy

**DOI:** 10.1371/journal.pone.0146787

**Published:** 2016-01-29

**Authors:** Jun-Fang Liao, Li Ma, Xiao-Jing Du, Mei Lan, Ying Guo, Lie Zheng, Yun-Fei Xia, Wei Luo

**Affiliations:** 1 Department of Radiation Oncology, Sun Yat-sen University Cancer Center, State Key Laboratory of Oncology in South China, Guangzhou 510060, Guangdong Province, People's Republic of China; 2 Department of Medical Statistics and Epidemiology, State Key Laboratory of Oncology in South China, Sun Yat-sen University Cancer Center, Guangzhou, People’s Republic of China; 3 Department of Imaging Department, State Key Laboratory of Oncology in South China, Sun Yat-sen University Cancer Center, Guangzhou, People’s Republic of China; Taipei Medical University, TAIWAN

## Abstract

**Purpose:**

To investigate the prognostic value of cavernoussinus invasion (CSI) in patients with nasopharyngeal carcinoma (NPC) treated with intensity-modulated radiotherapy (IMRT).

**Patients and Methods:**

Retrospective review of data from 1,087 patients with biopsy-proven, non-metastatic NPC. All patients were diagnosed using magnetic resonance imaging (MRI) scans and received IMRT as the primary treatment.

**Results:**

The incidence of cavernoussinus invasion in this cohort was 12.1%. In univariate analysis, 5-year overall survival (OS) (70.6% vs. 88.5%, P < 0.001) and distant metastasis-free survival (DMFS) (71.4% vs. 87.7%, P < 0.001), but not locoregional relapse-free survival (LRFS) (93.9% vs. 93.7%, P = 0.341), were significantly different between patients with and without cavernoussinus invasion. In the T4 subgroup, the 5-year OS, DMFS, and LRFS of patients with and without cavernoussinus extension were 70.6% vs. 81.9% (P = 0.011), 71.4% vs. 84.1% (P = 0.011), and 91.2% vs. 89.7% (P = 0.501), respectively. In multivariate analysis, cavernoussinus invasion was an independent prognostic factor for poorer OS (HR = 1.782; P = 0.013) and DMFS (HR = 1.771; P = 0.016), but not LRFS (HR = 0.632; P = 0.294). In patients with lymph node metastasis, the DMFS rates of patients with and without cavernoussinus invasion were significantly different (P < 0.001). Preliminaryanalysis indicated that neoadjuvant chemotherapy led to better DMFS and OS in patients with cavernoussinus invasion than concurrent chemotherapy or radiotherapy alone; however, the differences were not significant.

**Conclusions:**

In the IMRT era, cavernoussinus invasion remains a prognostic factor for poor DMFS and OS in NPC, even in patients with T4 disease.

## Introduction

Nasopharyngeal carcinoma (NPC) is a squamous cell carcinoma that is relatively common in Asia, especially individuals of Southern Chinese ethnicity [1]. NPC is radiosensitive and is mainly treated by radiotherapy (RT). Compared with two-dimensional conventional radiotherapy (2D-CRT), intensity-modulated radiation therapy (IMRT) has the advantages of better tumor coverage and normal organ sparing, which enables dose escalation. A series of studies have reported that the local control rates at 2–5 years for patients with NPC now exceed 90% in the IMRT era. However, distant metastasis remains a significant challenge to the treatment of NPC [2–4]. Therefore, early identification of factors associated with an increased risk of distant metastasis is an essential step towards improving the effectiveness of existing treatments.

Invasion into the venous plexus located in the parapharyngeal space and marrow of the bones in the skull base is considered a potential route for the spread of hematogenous disease in patients with NPC [5]. Chen Lei et al. reported that T4 category patients with involvement of the intracranial region, cranial nerves, and/or the orbit are more likely to develop distant metastases and death after IMRT than patients with involvement of the masticator space only [6]. Tsung-Min Hung et al. found that the intracranial region of the prepontine cistern invasion (PPCI) is an independent poor prognostic factor for DMFS in patients with NPC treated with IMRT, even within patients with T4 disease. These observations confirmed that the sub-classification of T4 disease has different prognostic value for patients with NPC [7].The cavernous sinus is a critical, highly-vascularized area region that contains a complex mixture of cranial nerves (CNs; III, IV, V1, V2 and VI), the internal carotid artery (ICA) and the venous plexus [8]. As NPC is highly invasive and metastatic, the disease can invade into this vascular sinus through the foramen ovale and foramen lacerum or directly via skull base erosion in patients with locally advanced tumors [9].However, to our knowledge, the prognostic value of cavernoussinus invasion (CSI) has not been examined in the IMRT era. According to the 7th edition of the American Joint Committee on Cancer (AJCC) staging system, intracranial involvement is defined as part of the T4 classification in NPC [10]. Even in the modern IMRT era, radiotherapy for patients with a T4 classification remains a great challenge, due to their high tumor load and the proximity of critical structures such as the spinal cord and brain stem to the tumor.

Magnetic resonance imaging (MRI) provides a superior soft tissue contrast that enables more accurate assessment of the intracranial area than computed tomography (CT) [11]. Therefore, we undertook this study to assess the prognostic value of CSI detected by MRI in a large cohort of patients with NPC treated with IMRT and to assess the most suitable position for CSI within the T classification system.

## Materials and Methods

### Patient selection and staging

Approval for retrospective analysis of the patient data was obtained from the ethics committee of Sun Yat-sen University Cancer Center. The Ethics committee of Sun Yat-sen University Cancer Center waived the need for written informed consent from the participants as this was a retrospective image-review study. All patient records were anonymized and de-identified prior to analysis. Between January 2004 and November 2009, a total of 1214 consecutive patients with newly-diagnosed, non-metastatic, biopsy-proven NPC received radical IMRT at our institution. Of these, 127 patients who were diagnosed by CT imaging were excluded and the remaining 1087 patients were retrospectively reviewed in this study. All patients were re-staged according to the 7th edition of the AJCC staging system [10]. Table 1 lists the characteristics of all patients in this study, and Table 2 summarizes the features of the patients with T4 NPC stratified by the presence or absence of CSI.

**Table 1:** Clinical features of the 1087 patients with NPC.

Characteristic No. of patients (%)	
Age (years)	
<50	788 (72.5%)
≥50	299 (27.5%)
Gender	
Male	805 (74.1%)
Female	282 (25.9%)
Histology (WHO category)	
I category	22 (2%)
II-III category	1065 (98%)
T-category^a^	
T1	125 (11.5%)
T2	221 (20.3%)
T3	423 (38.9%)
T4	318 (29.3%)
N-category^a^	
N0	217 (20.0%)
N1	482 (44.3%)
N2	305 (28.1%)
N3	83 (7.6%)
Clinical stage^a^	
Stage I	66 (6.1%)
Stage II	165 (15.2%)
Stage III	482 (44.3%)
Stage IVa	291 (26.8%)
Stage IVb	83 (7.6%)
Chemotherapy	
No	222 (20.4%)
Yes	865 (79.6%)

Abbreviation: WHO = World Health Organization; N = node; T = tumor; NPC = nasopharyngeal carcinoma; AJCC = American Joint Committee on Cancer; UICC = Union for International Cancer Control

^a^, According to the 7th edition of the UICC/AJCC staging system.

**Table 2:** Characteristics of the patients with T4 NPC stratified by cavernoussinus invasion.

Characteristic	T4 with CSI (n = 131)	T4 without CSI (n = 187)	P-value
	No. of patients (%)	No. of patients (%)	
Age (years)			
<50	92 (70%)	120 (64.2%)	0.259
≥50	39(30%)	67(35.8)%	
Gender			
Male	103 (81.7%)	134 (71.6%)	0.160
Female	28 (21.3%)	53 (28.4%)	
Histology			
WHO grade I	4 (3%)	1 (0.5%)	0.608
WHO grade II-III	128 (97%)	186 (99.5%)	
N-stage			
N0- N1	31 (23.7%)	78 (41.7%)	0.001
N2-N3	100 (76.3%)	109 (58.3%)	
Chemotherapy			
Yes	124(94.7%)	176 (94.1%)	0.838
No	7(5.3.%)	11 (5.9%)	

### Imaging protocols

All patients underwent MRI using a 1.5-Tesla system (Signa CV/i; General Electric Healthcare, Chalfont St. Giles, United Kingdom) using a head and neck coil. The scanning field of view ranged from the superior margin of the frontalsinus to the level of the supraclavicular fossa. T1-weighted fast spin-echo images in the axial, coronal and sagittal planes (repetition time 500–600 ms, echo time 10–20 ms) and T2-weighted fast spin-echo MR images in the axial plane (4000–6000 ms, echo time 95–110 ms) were obtained before injection of contrast material. After the intravenous administration of gadopentetate dimeglumine (Gd-DTPA; Magnevist, Schering, Berlin) at 0.1 mmol per kilogram/body weight, axial and sagittal T1-weighted spin-echo sequences and coronal T1-weighted fat-suppressed spin-echo sequences were performed sequentially using the same parameters as prior to the injection of Gd-DTPA. A section thickness of 3 mm was used for coronal sequences and 5 mm for other sequences.

### Image assessment

**Fig 1:**
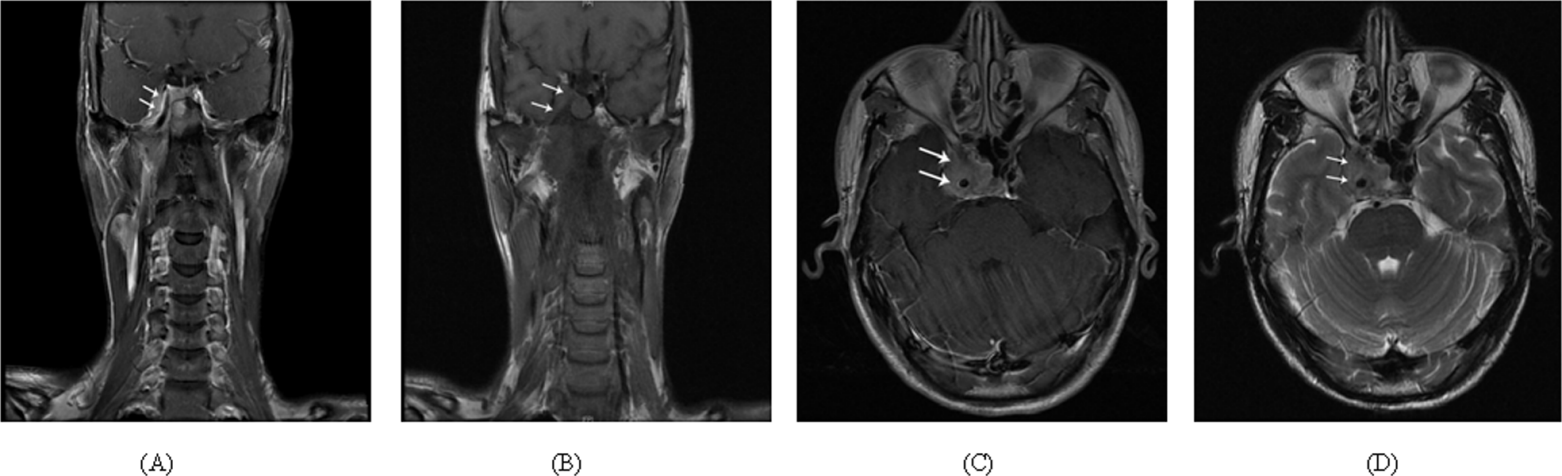
Representative appearance of cavernoussinus invasion (CSI) on multi-serial plane MRI scan images from an individual patient with NPC. (A) Enhanced coronal T1W fat-suppressed magnetic resonance (MR) image showing invasion of the right CS via the foramen lacerum (upward arrow). (B) Plain coronal T1W MR image of the same scanning plane as shown in (A). (C) The lesion is seen to extend to the right CS (arrow) and the orbital apex on the axial T1W MR image. (D) Axial T2W MR image of the same scanning plane as shown in (C).

Two radiologists each with more than 10 years of experience in head and neck cancer MRI evaluated all scans separately; any disagreements were resolved by consensus. In general, NPC spreads into the cavernoussinus via three routes. The most frequent route is through the foramen ovale via perineural spread, with less frequent invasion through the foramen lacerum. Intracranial extension may also occur directly via skull base erosion [9]. The typical MR imaging characteristics of CSI are an enlarged cavernoussinus with contrast enhancement and tumor development inside the cavernoussinus. NPC appears hypointense on T1W and T2W images. Contrast enhancement is moderate or prominent and usually inhomogeneous [12], as shown in Fig 1.

### Treatment

All patients underwent radical radiation therapy. Thennasopharyngeal and upper neck tumor volumes were treated with IMRT for the entire treatment course. A conventional anterior or anteroposterior opposing cervical technique was used for the lower neck. All targets were treated simultaneously using the simultaneous integrated boost (SIB) technique. The median dose prescribed at GTVnx was 68 (range 68–72) Gy in patients presented with CSI and 68 (range 68–75) Gy in patients presented without CSI, respectively. Both of the two groups were in 29–33 fractions. Non-statistically significant difference was observed in terms ofprescribed dose of GTVnx between the two groups (p = 0.518).Details of the techniques used at Sun Yat-sen University Cancer Center have been reported previously [13].

During the study period, our institutional chemotherapy guidelines were based on the 6th edition of the UICC/AJCC staging system. A total of 92.7% (794/856) of the patients with stage III or IV disease in this study had received chemotherapy. The chemotherapy regimens included cisplatin-based concurrent chemotherapy alone, concurrent chemotherapy combined with neoadjuvant chemotherapy and/or adjuvant chemotherapy. However, 5.02% (43/856) patients with a severe underlying disease could not tolerate chemotherapy, 0.35% (3/856) patients refused chemotherapy and 1.86% (16/856) patients restaged from category II to category III received radiotherapy alone.When possible, salvage treatments such as intracavitary brachytherapy, surgery and chemotherapy were provided in the event of documented relapse or persistent disease.

### Follow-up and statistical analysis

Pass version 11.0 (NCSS, LLC. Kaysville,Utah, USA) were used for sample size estimation. According to Liu et al[14], approximately 9.8% patients initially present with cranial nerve palsy. The proportion of patients with cavernous sinus involvement was taken as 10%, the power was set at 0.95, and the significance level was set at 0.05. The proportion lost to follow-up was about 10%, the proportion of patients surviving without CSI was 85%, and the proportion of patients surviving with CSI was taken as 65%. The result of the power calculation was a sample size of 919 patients.

All analyses were performed using SPSS version 19.0 (IBM Corporation, Armonk, NY, USA). Follow-up duration was calculated from the first day of diagnosis to either the day of death or last follow-up. Follow-up was based on imaging or clinical assessment. Patients were examined at least every 3 months during the first 2 years, and thereafter, a follow-up examination was performed every 5 months during years 3–5 or until death. Locoregional recurrence was assessed by MRI and nasopharyngoscopy. Patients with residual or recurrent local disease underwent biopsy to confirm malignancy. Distant metastasis was assessed via chest X-ray, ultrasonography of the abdomen and a whole-body bone scan. In addition, quantification of plasma Epstein-Barr virus (EBV) deoxyribonucleic acid (DNA) was employed as an important indicator of recurrence and metastasis in NPC during the duration of follow-up.

The end-points (time to the first defining event) used were the overall survival (OS), the distant metastasis-free survival (DMFS) and the local relapse-free survival (LRFS). The Kaplan-Meier method was used to estimate survival [15], and differences were compared using the log-rank test [16]. The chi-square test was used to compare the clinical features of patients with T4 NPC with and without cavernoussinus invasion. To assess for independent significance, multivariate analyses was performed using the Cox proportional hazards model with a backwards elimination of insignificant explanatory variables [17]. Statistical significance was set at α = 0.05 and P-values were determined using two-sided tests.

## Results

### Incidence of cavernoussinus invasion

MRI images for 1087 patients with NPC were retrospectively reviewed in this study. According to the 7th edition of the TNM(Tumor Node Metastasis) staging system, 66/1087 (6.1%) patients had clinical stage I NPC, 165/1087 (15.2%) had stage II, 482 /1087(44.3%) had stage III, and 374 /1087(34.4%) had stage IV NPC (291 stage IVA, 83 stage IVB). The median age for the entire cohort was 43 (range, 11–86 years); 805 (74.1%) patients were male and 282 (25.9.7%) were female (Table 1). Overall, 131/1087 (12.1%) patients in this series had CSI; the incidence of CSI in patients with T4 NPC was 41.2% (131/318; Tables 1 and 2).

### Prognostic value of cavernoussinus invasion in the entire cohort

**Fig 2:**
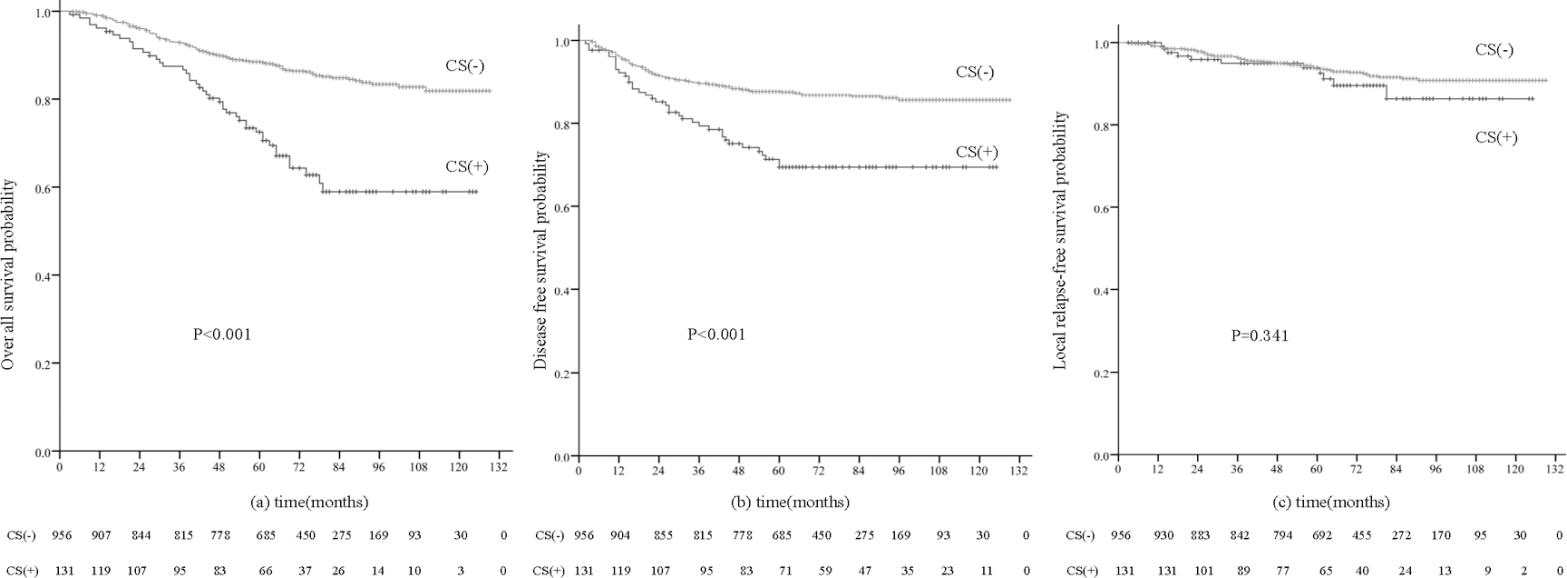
(a) Overall, (b) distant metastasis-free and (c) local relapse-free survival curves for all 1087 NPC patients with and without cavernoussinus invasion (CSI).

The median follow-up period was 70 months (range, 3–129 months). The percentage of patients lost to follow-up in the study was 10.4% (113/1087). These were censored in the Kaplan-Meier analysis.By the end of the follow-up period, 79/1087 (7.3%) patients had suffered local-regional failure, 160 (14.7%) had developed distant metastases, 21 (1.9%) had developed both locoregional recurrence and distant metastases and 178 patients (16.4%) had died. For the entire cohort, the 5-year OS, DMFS and LRFS rates were 83.6%, 85.3% and 92.7% respectively. In univariate analysis, the 5-year OS (70.6% vs. 88.5%, P < 0.001) and DMFS (71.4% vs. 87.7%, P < 0.001) rates of patients with and without CSI were significantly different; however, LRFS (93.9% vs. 93.7%, P = 0.341) was not significantly different between groups (Fig 2).

The following parameters were included as covariates in multivariate analysis of all patients: age (<50 vs. ≥50 years), gender (female vs. male), pathology category (keratinizing vs. non-keratinizing); nasal cavity extension, oropharyngeal extension, parapharyngeal space extension, skull base erosion, paranasal sinus extension, hypopharyngeal extension, masticator space extension, cranial nerve palsy, CSI, intracranial invasion (intracranial invasion with the expectation of cavernous sinus invasion, such as dural invasion, brain tissue invasion and prepontine cistern invasion), N category (N0-1 vs. N2-3), chemotherapy (none vs. yes). As is shown in Table 3,CSI was found to be a significant independent prognostic factor for OS and DMFS, but not LRFSin multivariate analysis. In addition, unadjustedresults were summarized in Table 4.

**Table 3:** Multivariate analyses of prognostic factors for all 1087 patients with NPC.

Endpoint	Variable	HR	P-value	95% CI for HR
OS	Cavernoussinus invasion	1.782	0.013	1.129–2.813
	N category	3.023	<0.001	2.218–4.118
	Gender	1.811	0.003	1.221–2.685
	Age	1.877	<0.001	1.380–2.554
	Masticator space invasion	1.433	0.038	1.018–2.018
	Cranial nerve invasion	1.727	0.035	1.039–2.869
	Chemotherapy	0.617	0.034	0.369–0.963
	Skull base invasion	2.347	<0.001	1.543–3.589
DMFS	Cavernoussinus invasion	1.771	0.016	1.14–2.816
	N category	2.621	<0.001	1.912–3.592
	Gender	1.571	0.021	1.071–2.309
	Skull-base invasion	2.286	0.075	1.490–3.505
	Cranial nerve invasion	1.613	0.075	0.953–2.732
LRFS	Cavernoussinus invasion	0.294	0.632	0.269–1.488
	Masticator space invasion	1.898	0.012	1.151–3.131
	Nasal cavity invasion	1.622	0.057	0.987–2.666
	N category	1.532	0.063	0.977–2.401
	Cranial nerve invasion	1.852	0.074	0.941–3.644

Abbreviation: NPC = nasopharyngeal carcinoma; OS = overall survival; DMFS = distant metastasis-free survival; LRFS = locoregional relapse-free survival; CI = confidence interval HR = hazard ratio; P-values were calculated using an adjusted Cox proportional hazards model.

**Table 4:** Hazard ratio and significance (P) in an unadjusted model for all 1087 patients with NPC.

Endpoint	Variable	HR	P-value	95% CI for HR
OS	Cavernoussinus invasion	2.772	<0.001	1.971–3.899
	N category	2.323	<0.001	1.804–3.256
	Masticator space invasion	2.206	<0.001	1.612–3.019
	Cranial nerve invasion	3.189	<0.001	2.162–4.702
	Skull-base invasion	2.722	0.075	1.838–4.031
	Cranial nerve invasion	1.613	0.075	0.953–2.732
DMFS	Cavernoussinus invasion	2.423	<0.001	1.678–3.499
	N category	2.390	<0.001	1.752–3.260
	Masticator space invasion	1.408	0.063	0.982–2.020
	Cranial nerve invasion	2.615	<0.001	1.706–4.009
	Skull base invasion	2.707	<0.001	1.790–4.093
LRFS	Masticator space invasion	2.131	0.002	1.326–3.424
	Cavernoussinus invasion	1.361	0.343	0.719–2.575
	N category	1.504	0.074	0.961–2.353
	Cranial nerve invasion	2.411	0.007	1.274–4.565

### Prognostic value of cavernoussinus invasion in T4 NPC

**Fig 3:**
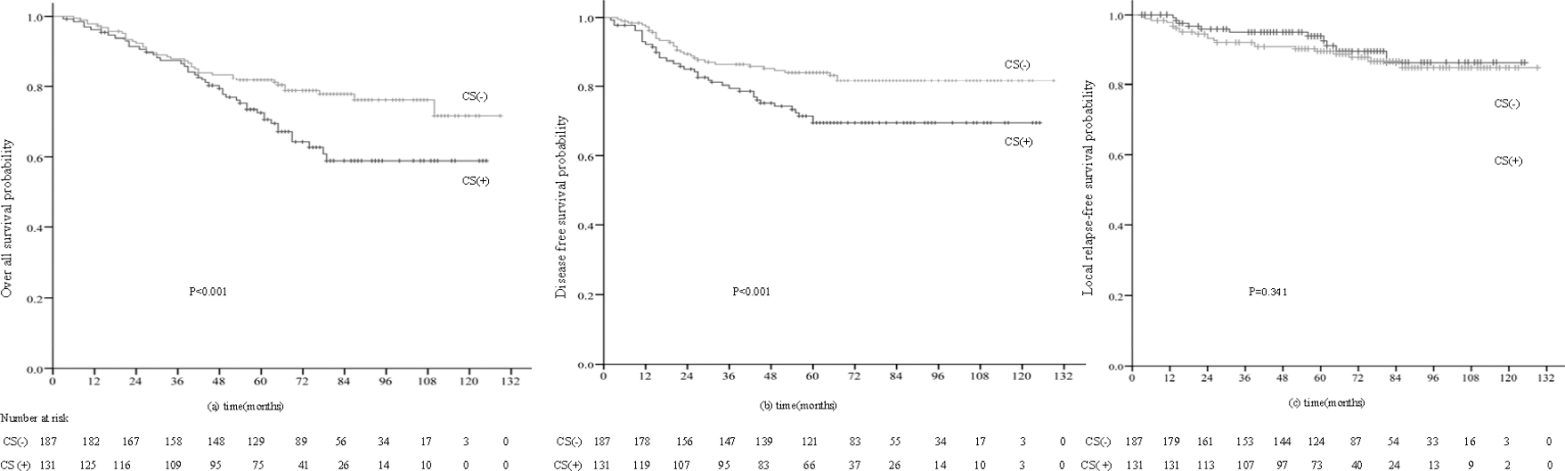
(a) Overall, (b) distant metastasis-free and (c) local relapse-free survival curves for the 318 patients with T4 NPC with and without cavernoussinus invasion (CSI).

In T4 NPC, the 5-year OS, DMFS and LRFS rates for patients with and without CSI were 70.6% vs. 81.9% (P = 0.011), 71.4% vs. 84.1% (P = 0.011) and 91.2% vs. 89.7% (P = 0.501), respectively (Fig 3).

In a similar manner to the analysis for the entire cohort, multivariate analysis was performed to adjust for various prognostic factors in the patients with T4 NPC. The parameters included in the Cox proportional hazards model were age, gender, pathology category, hypopharyngeal extension, masticator space extension, cranial nerve palsy, CSI, intracranial invasion, N category (N0-1 vs. N2-3), and chemotherapy (none vs. yes). CSI was an independent prognostic factor for OS (HR, 1.675; 95% CI, 1.029–2.724; P = 0.038) and DMFS (HR, 1.827; 95% CI, 1.076–3.100; P = 0.026) in patients with T4 NPC, but not LRFS (HR, 0.775; 95% CI, 0.334–1.796; P = 0.552) (Table 5).

**Table 5:** Multivariate analyses of prognostic factors for the 318 patients withT4 NPC.

Endpoint	Variable	HR	P-value	95% CI for HR
OS	Cavernoussinus invasion	1.675	0.038	1.029–2.724
	N stage	2.295	<0.001	1.456–3.618
	Age	2.042	0.002	1.308–3.188
	Cranial nerves invasion	1.640	0.053	0.993–2.706
	Gender	1.789	0.051	0.997–3.212
LRFS	N category	1.872	0.074	0.942–3.722
	Cavernoussinus invasion	0.775	0.552	0.334–1.796
DMFS	Cavernoussinus invasion	1.762	0.034	1.043–2.977
	N category	2.447	<0.001	1.505–3.979
	Gender	1.979	0.033	1.057–3.705
	Cranial nerves invasion	1.605	0.083	0.941–2.738

### Cavernoussinus invasion and lymph node metastasis

**Fig 4:**
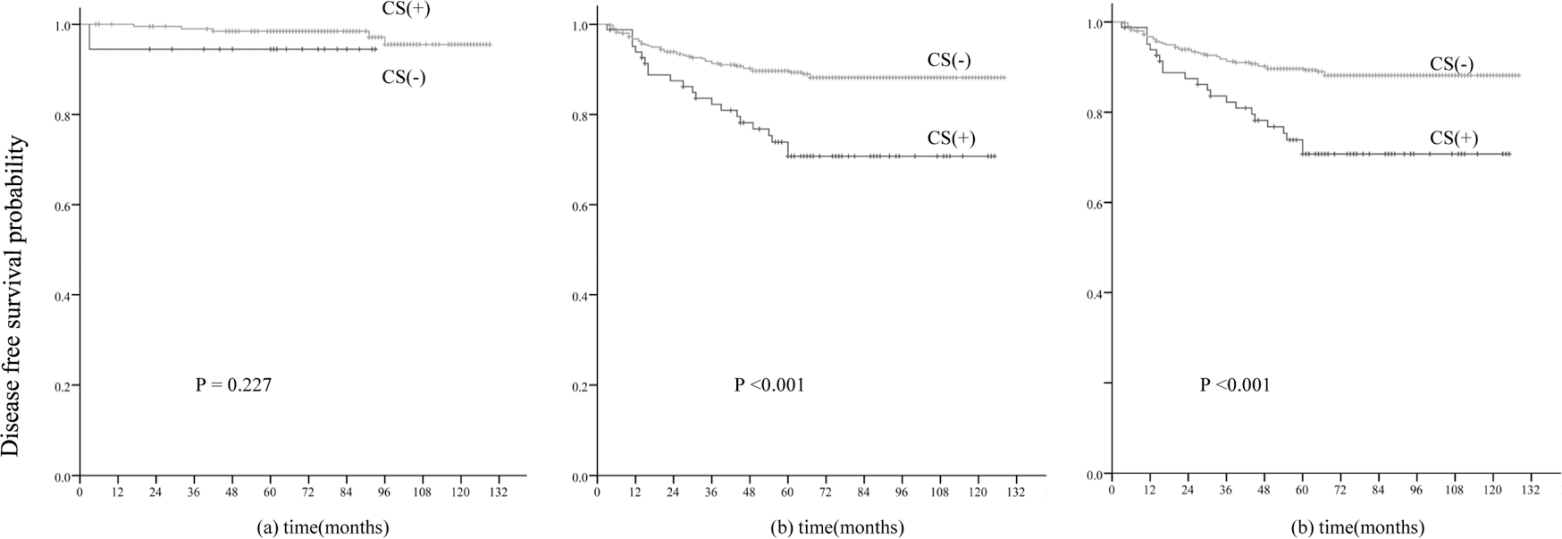
Distant metastasis-free survival curves for all 1087 patients with NPC with and without cavernoussinus invasion (CSI) stratified by regional lymph node metastasis. (a)Group 1: N0 disease; (b) Group 2: N1 disease;(c)Group 3: N2-N3 disease.

To further evaluate the impact of CSI and lymph node metastasis on prognosis in NPC, we divided all 1087 patients into three groups according to N category. Group 1 included the 217 patients with N0 disease, Group 2 included the 482 patients with N1 disease and Group 3 included the 388 patients with N2-N3 disease. As is shown in Fig 4, the DMFS rates of patients with and without CSI were significantly different in Group 2 (N1 disease, P < 0.001) and Group 3 (N2-N3 disease; P < 0.001), but not in Group 1 (N0 disease; P = 0.227). In multivariate analysis, clinical variables (age, gender, pathology, T category, chemotherapy, and CSI) were included in the Cox proportional hazards model. CSI was found to be a significant independent factor for DMFS with N1 (HR, 2.577; 95% CI, 1.546–4.297; P < 0.001) and N2-N3 (HR, 2.240; 95% CI, 1.251–4.008; P < 0.001) disease.

### Chemotherapy in patients with cavernoussinus invasion

**Fig 5:**
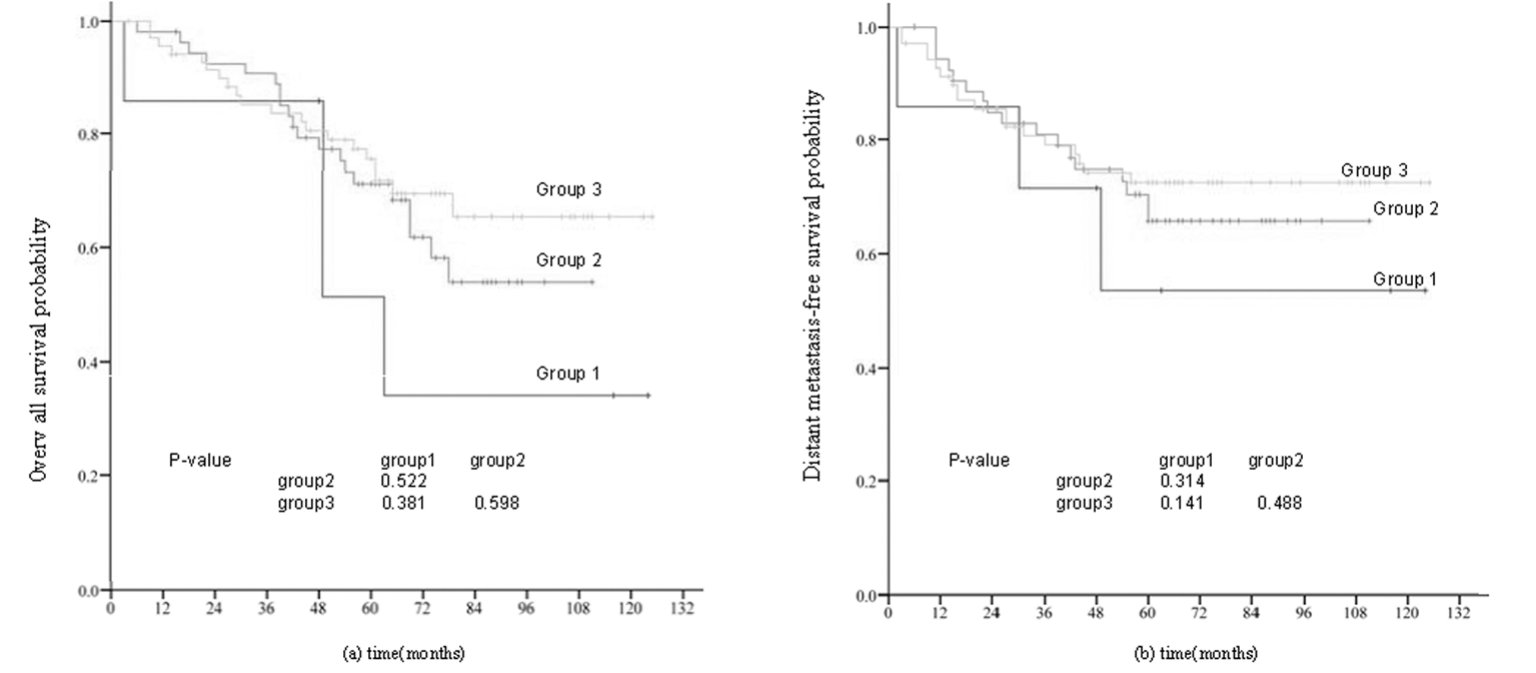
(a) Overall and (b) distant metastasis-free survival curves for 131 NPC patients with cavernoussinus invasion (CSI) stratified by chemotherapy regimen. Group 1: radiotherapy alone; Group 2: concurrent chemoradiation (CCRT); Group 3: neoadjuvant chemotherapy (NACT) + CCRT or NACT.

Clinical trials and systematic reviews have demonstrated concurrent chemoradiotherapy (CCRT) is the most effective treatment for locally advanced NPC, and CCRT with or without adjuvant chemotherapy remains the standard treatment for locally advanced NPC [18–19]. Neoadjuvant chemotherapy (NACT) administered prior to CCRT can reduce the size of the primary tumor and eradicate the micro-metastatic tumor burden. However, the value of NACT remains controversial. Therefore, we assessed the prognostic value of NACT in patients with CSI. The 131 patients with CSI were divided into three groups based on whether they received NACT or not. Group 1 included the seven patients who received radiotherapy alone, Group 2 included the 45 patients who received CCRT and Group 3 included the 79 patients who received NACT or NACT + CCRT. The OS and DMFS rates of Group 1, 2 and 3 were 58.1% vs. 68.5% vs. 74.3% and 42.9% vs. 63.0% vs. 71.4%, respectively; however, the differences between groups were not statistically significant (Fig 5).

### Late toxicity of patients with cavernous sinus involvement after IMRT

The limit dose of optic nerve and chiasmawere both 50 Gy in patients with cavernous sinus invasion, while the mean maximum doses of the left and the right optic nerve were 55.51 Gy and 55.39 Gy respectively.However, only 21 patients had delineated the optic chaisma and the mean maximum dose of the 21 patients was 58.87 Gy.At the last follow-up visit, late toxicities such as otological toxicities, xerostomia, trismus, subcutaneous tissue fibrosis, visual impairment, radiation encephalopathy were observed Finally, 14.3% (15/105) patients with cavernous sinus involvement presented with visual impairment, and 2.85% (3/105) patients among them were completely blind (Table 6).

**Table 6:** Late toxicities of patients with cavernous sinus involvement after IMRT.

Toxicity	Grade					
	N	0	1	2	3	4
Trismus	104	86 (82.7%)	12 (11.5%)	6 (5.8%)	0 (0%)	0 (0%)
Otologic toxicities	106	43 (40.6%)	49 (46.2%)	14 (13.2%)	0 (0%)	0 (0%)
Visual impairment	105	90 (85.7%)	5 (4.8%)	4 (3.8%)	3 (2.85%)	3 (2.85%)
Xerostomia at 12 months after IMRT	106	2 (1.9%)	52 (49%)	48 (45.3%)	4 (3.8%)	0 (0%)
Xerostomia at thelast follow-up	105	13 (12.6%)	76 (73.8%)	15 (14.6%)	0 (0%)	0 (0%)
Subcutaneoustissue fibrosis	105	24 (22.9%)	76 (72.4%)	6 (5.7%)	0 (0%)	0 (0%)
Radiation encephalopathy	103	76 (73.8%)	25 (24.3%)	2 (1.9%)	0 (0%)	0 (0%)

## Discussion

Brigette et al. [20]. reported that CSI had minimal impact on the treatment outcome of patients with locoregionally advanced NPC treated with two-dimensional radiotherapy. In analysis of a small cohort, it possible that the effect of tumor vascular invasion in locoregionally advanced disease may have been concealed by other factors, such as disease stage and treatment. In contrast, the current large cohort study revealed that CSI was an independent prognostic factor for both OS and DMFS in all patients, as well as patients with T4 disease. The distinct anatomic structure and function of the cavernoussinus may explain the relationship between CSI and a poor prognosis. The cavernoussinus contains a complicated and crucial network that consists of the carotid artery, venous plexus and cranial nerves, which communicate in a complicated manner with various paracavernous venous channels. Posteroinferiorly, the cavernoussinus communicates with the inferior petrosal sinus and drains into the internal jugular vein. Extracranially, the cavernoussinuscommunicates with the pterygoid venous plexus and drains into the deep facial vein. The bilateral cavernoussinuses are interconnected with each other at the midline via the small anterior and posterior intercavernous sinuses and the basal venous plexus [8]. Patients with NPC who have CSI usually have extensive invasive disease accompanied by bone destruction; thus, it is reasonable to expect an increased likelihood of hematogenous metastasis in patients with CSI.

Although IMRT can provide excellent locoregional control in locally advanced NPC, distant metastasis still remains the most difficult challenge in the treatment of NPC [21]. In this study, the 5-year LRFS rate for patients with T4 disease was 89.6%; however, the DMFS rate was only 78.6%. One approach to reduce these modes of treatment failure would be to identify patients at high risk of distant metastasis in order to explore more effective systemic treatment strategies for these patients. Several factors have been linked to an increased risk of distant recurrence, for instance, Feng et al. [22]. reported that patients with a primary tumor volume > 40 ml had significantly lower 5-year LRFS and DMFS rates. In addition, the circulating EBV DNA level correlates significantly with the tumor load, TNM stage and recurrence and survival rates in NPC [23–24]. It is worth mentioning that this study identified CSI as a significant independent prognostic factor for OS and DMFS in patients with advanced stage (T4) NPC; this result also serves to remind us of the varied prognosis of patients with different sub-types of T4 NPC.

As the main advantage of NACT is to eradicate distant micrometastases and decrease the tumor volume before radiation, the addition of NACT to CCRT may be a reasonable approach to improve prognosis. Hui et al. [25] reported NACT using a docetaxel/cisplatin regimen provided a survival benefit in locally advanced NPC when administered in addition to CCRT with weekly cisplatin. However, Tan et al. [26] reported that the addition of cisplatin-based induction chemotherapy to RT did not improve OS. One plausible explanation for these conflicting findings is the inability to properly classify patients with different risk profiles during trial enrollment. In this study, we found that 5-year OS and DMFS were better in the NACT group than the CCRT group; however, the differences between groups were not significant. It is possible that this study was not large enough to attain statistical significance. Discovery of a factor that identifies patients at high risk of distant metastasis may help to enhance the efficacy of NACT + CCRT. Further studies are required to explore the impact of NACT in the sub-group of patients with T4 disease and CSI.

The presence of cervical lymph node metastasis is clearly established as an adverse prognostic factor for distant metastasis in NPC. In this study, the incidence of distant metastasis in the N0, N1, N2, and N3 category groups was 2.8%, 13.7%, 21.6%, and 26.5%, respectively. Further analysis revealed that CSI significantly negatively affects DMFS in patients with N1 and N2-N3 disease, but not in the N0 group. The nasopharynx has a well-developed network of lymphatics and the normal lymph nodes are important for the proper functioning of the immune system, acting as filters for foreign particles and cancer cells. Therefore, in patients with N0 disease, the lymph nodes function normally to prevent CSI developing into distant metastasis. However, once the normal structure of the lymph nodes is destroyed by cancer cells, the pathological lymph nodes may not be able to execute normal immune function and prevent metastasis.

This study indicates that CSI does not have a significant effect on LRFS in NPC. This can mainly be attributed to the following factors. Firstly, with the aid of MRI, the range of tumor infiltration can be evaluated with greater accuracy and the target volume can be designed more precisely [27]. Additionally, recent improvements in treatment strategies for locally advanced NPC, including IMRT and the combination of chemotherapy with radiotherapy, have dramatically improved treatment outcomes with respect to loco-regional control [28].

The major limitation of this study is its retrospective nature. Nevertheless, this report is noteworthy as it is the first assessment of the prognostic value of CSI in a large number of patients with NPC who were diagnosed with MRI, treated with IMRT and for whom long-term follow-up was available.

## Conclusion

In conclusion, CSI confers a poorer prognosis in terms of both DMFS and OS in patients with NPC treated with IMRT, even in the T4 category group. More importantly, this study provides additional data to help identify subsets of patients with T4 disease at high risk of distant failure, which may enable more aggressive systemic treatments to be tested.

## Supporting Information

S1 File Primary data of each analyze. (XLS)
